# The role of Sema3–Npn-1 signaling during diaphragm innervation and muscle development

**DOI:** 10.1242/jcs.186015

**Published:** 2016-09-01

**Authors:** Maximilian Michael Saller, Rosa-Eva Huettl, Philipp Hanuschick, Anna-Lena Amend, Paolo Alberton, Attila Aszodi, Andrea B. Huber

**Affiliations:** 1 Experimental Surgery and Regenerative Medicine, Department of Surgery, Ludwig-Maximilians-University (LMU), Nußbaumstraße 20, Munich 80336, Germany; 2 Institute of Developmental Genetics, Helmholtz Zentrum München – German Research Center for Environmental Health (GmbH), Ingolstädter Landstraße 1, Neuherberg 85764, Germany; 3 Institute of Physiology, Department of Physiological Genomics, Ludwig-Maximilians-University (LMU), Schillerstraße 46, Munich 80336, Germany; 4 Bernstein Network for Computational Neuroscience, Albert-Ludwigs-University, Freiburg, Germany

**Keywords:** Axon pathfinding, Diaphragm, Muscle development, Npn-1, Phrenic nerve, Sema3a

## Abstract

Correct innervation of the main respiratory muscle in mammals, namely the thoracic diaphragm, is a crucial pre-requisite for the functionality of this muscle and the viability of the entire organism. Systemic impairment of Sema3A–Npn-1 (Npn-1 is also known as NRP1) signaling causes excessive branching of phrenic nerves in the diaphragm and into the central tendon region, where the majority of misguided axons innervate ectopic musculature. To elucidate whether these ectopic muscles are a result of misguidance of myoblast precursors due to the loss of Sema3A–Npn-1 signaling, we conditionally ablated *Npn-1* in somatic motor neurons, which led to a similar phenotype of phrenic nerve defasciculation and, intriguingly, also formation of innervated ectopic muscles. We therefore hypothesize that ectopic myocyte fusion is caused by additional factors released by misprojecting growth cones. Slit2 and its Robo receptors are expressed by phrenic motor axons and migrating myoblasts, respectively, during innervation of the diaphragm. *In vitro* analyses revealed a chemoattractant effect of Slit2 on primary diaphragm myoblasts. Thus, we postulate that factors released by motor neuron growth cones have an influence on the migration properties of myoblasts during establishment of the diaphragm.

## Abstract (precis)

**Highlighted Article:** Sema3A–Npn-1 signaling cell autonomously influences phrenic nerve branching in the diaphragm and induces ectopic muscle formation through secondary mechanisms, possibly by Slit–Robo signaling.

## INTRODUCTION

Faithful establishment of sensory motor circuitry and neuro-muscular connectivity is a crucial pre-requisite for the viability of all higher organisms. Peripheral axons leave the motor columns situated along the rostro-caudal axis of the spinal cord as segment-specific spinal nerves that undergo re-sorting into distinct fasciculated branches that are guided precisely towards their respective target muscles. Over the past decades, several ligand–receptor-based signaling systems have been discovered that tightly regulate and organize this targeted outgrowth of motor axons towards their appropriate targets. Subsequently, a special focus has been put on the sophisticated innervation of the extremities during embryonic development, involving accurate dorsal-ventral guidance decisions of limb-innervating axons that originate from motor neurons situated in the lateral motor columns at the brachial and lumbar levels of the spinal cord (Dasen et al., 2008; Tsuchida et al., 1994). In this regard, the ephrin–Eph signaling pathway, among others, is crucially involved in guidance decisions of motor axons originating in the medial or lateral aspects of the lateral motor column to precisely target ventral (ephrinB–EphB1) and dorsal (ephrinA–EphA4) limb musculature, respectively (Helmbacher et al., 2000; Wang and Anderson, 1997). Expression of certain guidance molecules, such as the receptor for secreted class 3 semaphorin (Sema3) F, namely neuropilin 2 (*Npn-2*, also known as *Nrp2*), corroborates correct pathfinding of developing motor axons in a subset-specific manner only in neurons of the medial part of the lateral motor column (Huber et al., 2005). *Npn-1* (also known as *Nrp1*), a close relative of *Npn-2*, is involved in peripheral axon guidance in a more widespread manner; its high affinity to Sema3A enables fasciculated growth of both motor and sensory axons in a cell autonomous manner, which most likely is governed by repulsive interactions between axons and the surrounding tissues in limbs during embryonic development. Furthermore, timing and coupling of sensory axon growth to motor axon extension crucially depend on expression of Npn-1 on sensory fibers (Huber et al., 2005; Huettl et al., 2011).

Although targeted innervation of the extremities has been studied extensively, initial targeting and fasciculated innervation of the thoracic diaphragm, one of the most essential respiratory muscles in all mammals, is only poorly understood. The highly patterned process of breathing is controlled by automatic respiratory centers in the brainstem or from the cerebral cortex which can, at least temporarily, override the automatic centers (Guz, 1997). These centers in the brainstem signal bilaterally to a specialized group of motor neurons in the cervical spinal cord, particularly motor neurons in the phrenic motor column (PMC) that constitute the phrenic nerves that innervate the diaphragm muscles. Injuries or malformations of these nerves lead to acute respiratory impairments (Burgess et al., 1989). Therefore, faithful innervation by the phrenic nerves is a requirement for the functionality of the highly specialized diaphragm and thus, ultimately, for the viability of the entire organism. Retrograde labeling experiments have revealed that phrenic motor neurons within the cervical spinal cord are demarcated by the expression of several transcription factors, most notably Hoxa5, Hoxc5, Isl1 and Scip (also known as Pou3f1), whereas they exclude the transcription factor FoxP1, which labels limb-innervating motor neurons (Bermingham et al., 1996; Dasen et al., 2008; Philippidou et al., 2012). Indeed, mice in which *Hoxa5* and *Hoxc5* are ablated show a severe loss of Scip-positive phrenic motor neurons, whereas genetic elimination of *FoxP1* induces a fate change of brachial motor neurons to acquire phrenic motor neuron characteristics (Dasen et al., 2008; Philippidou et al., 2012).

Although the timecourse of diaphragm development and its innervation by phrenic motor axons is well established, the molecular mechanisms that govern initial targeting of the diaphragm, as well as correct fasciculation and branching of the phrenic nerves are still to be elucidated. Well-known members of axon guidance cue families, such as Slit2 and its receptors Robo1 and Robo2 have been shown to be involved not only in targeting of the phrenic nerves towards the pleuroperitoneal folds (PPFs), from which the diaphragm muscle arises, but also in phrenic nerve fasciculation and innervation of the diaphragm musculature (Jaworski and Tessier-Lavigne, 2012). Both the receptor tyrosine kinase ErbB2, and Unc5c, a netrin receptor, were proven essential for the stabilization of neuro-muscular junctions (NMJs) or appropriate innervation of the costal muscle proportion, respectively (Burgess et al., 2006; Lin et al., 2000). *Npn-1* has been shown to be expressed in spinal motor neurons of the lateral motor column as well as medially positioned motor neurons in the ventral horn of the spinal cord (medial motor column), meaning that expression of *Npn-1* in at least a proportion of phrenic motor neurons is highly possible.

Here, we employed a genetic approach to investigate the involvement of the Sema3–Npn-1 signaling pathway in phrenic nerve targeting and fasciculated growth during the establishment of the diaphragm. We found that impairing this signaling pathway, either by mutation of the receptor so that secreted class 3 semaphorins can no longer bind to Npn-1 (*Npn-1^Sema−/−^*), or by conditional ablation of *Npn-1* in motor neurons by cell-specific activity of Cre-recombinase in motor neuron progenitor cells (*Npn-1^cond−/−^;Olig2-Cre*^+^), does not affect initial fasciculation and targeting of the phrenic nerves. However, it inflicts severe defasciculation of axons during innervation of the developing diaphragm muscle at later embryonic stages. Interestingly, in both mutant mouse lines, we observed formation of ectopic muscle patches within the normally muscle-free central tendon region of the diaphragm. Given that ectopic muscle development also occurred upon motor-neuron-specific ablation of *Npn-1*, we asked what triggers their formation, if not mis-migration of muscle progenitors due to the loss of systemic Sema3–Npn-1 signaling.

In *Drosophila*, tendon progenitor cells attract Robo-expressing muscle progenitors, through the release of Slit, towards the muscle attachment sides (Kramer, 2001). Furthermore, Slit–Robo signaling is involved in myoblast migration of medial musculature during chicken development (Halperin-Barlev and Kalcheim, 2011). We therefore investigated expression of Slit family members within Scip-positive phrenic motor neurons, as well as *Robo1* and *Robo2* expression in muscle progenitors during diaphragm innervation by the phrenic nerves. Furthermore, chemotaxis experiments with primary muscle progenitors of costal diaphragm muscles revealed that a subpopulation of primary muscle progenitors is attracted by Slit1 or Slit2. These results thus suggest an involvement of Slit–Robo signaling during muscle development and later innervation by somatic motor neurons, possibly by providing a condensation target during myofiber hypertrophy.

## RESULTS

### Npn-1 is expressed in motor neurons of the PMC during PPF targeting and diaphragm innervation

**Fig. 1.**
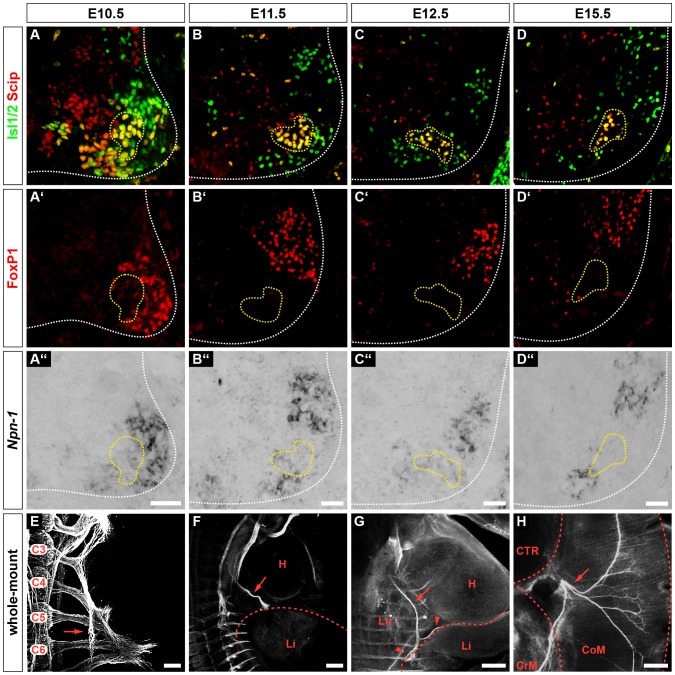
**Phrenic motor neurons express *Npn-1* during innervation of the thoracic diaphragm.** Phrenic motor neurons express Scip (red) and Isl1 or Isl2 (Isl1/2, green) and are negative for FoxP1 (red) (A–D,A′–D′). During phrenic nerve outgrowth and innervation of the diaphragm, a subpopulation of Scip^+^, Isl1^+^ and FoxP1^−^ motor neurons expresses *Npn-1* (A″–D″, yellow dotted line). At E10.5, phrenic nerves start to fasciculate from cervical spinal nerves at the levels C3–C5 within the brachial plexus (E, arrow). At E11.5, phrenic nerve projections (F, arrow) have formed a distinct bundle exiting the brachial plexus and target the developing diaphragm (F, dotted line). At E12.5, the phrenic nerve (arrow) branches into three distinct rami (G, arrowheads) that later innervate the costal and crural diaphragm musculature (H, arrow). H, heart; Li, liver; Lu, lung, CTR, central tendon region; CrM, crural muscle; CoM, costal muscle. Sagittal view (E–G), top view (H). Scale bars: 200 µm.

*Npn-1* is strongly expressed in motor neurons of the lateral motor columns at brachial and lumbar levels, as well as in neurons of the medial motor column (Huber et al., 2005; Huettl et al., 2011). *Npn-1^Sema−/−^* and *Npn-1^cond−/−^;Olig2-Cre^+^* mutant embryos are born according to Mendelian inheritance (Gu et al., 2003) (our observations); however, some newborns appear to be cyanotic and die within the first postnatal week. Therefore, in addition to the de-organized intercostal innervation, phrenic projections might also be affected in absence of Sema3–Npn-1 signaling (Huber et al., 2005; Huettl et al., 2011). To analyze whether *Npn-1* is expressed in phrenic motor neurons during initial targeting of the PPFs and later innervation of the developing muscle, we performed *in situ* hybridization against *Npn-1* and fluorescent immunohistochemistry against Scip, Isl1 and FoxP1 to localize motor neurons of the PMC (Fig. 1; Fig. S1) at crucial developmental stages (Philippidou et al., 2012).

In wild-type embryonic day (E)10.5 embryos, spinal nerves that contribute to the brachial plexus had converged at the base of the forelimb, and a distinct branch that will project caudally towards the PPFs had already formed on both sides of the developing embryo (Fig. 1E, arrow). One day later, the phrenic nerves had further extended between the developing heart and lungs and had almost reached the PPFs (Fig. 1F, red dotted line). By E12.5, the phrenic nerves had reached the PPFs and started to project dorsally and ventrally within the developing diaphragm (Fig. 1G, arrowheads). During the timecourse of initial diaphragm targeting, a subset of Scip^+^/Isl1^+^ (Fig. 1A–D, yellow dotted line) motor neurons in the PMC expressed *Npn-1* (Fig. 1A″–D″, yellow dotted line). Quantification of the positive *in situ* hybridization cells showed that 69.87±1.66% (mean±s.e.m.) of phrenic motor neurons expressed *Npn-1* at E10.5 and the numbers increased slightly to 74.83±1.38% at E11.5, and 77.68±1.17% at E12.5. By E15.5, crural and costal diaphragm muscle development and innervation were mostly established (Fig. 1H). Interestingly, at E15.5 the subpopulation of *Npn-1*-expressing phrenic motor neurons was noticeably, but not significantly, reduced to 55.60±3.71% (*P*=0.01) during diaphragm innervation (Fig. 1D–D″).

Taken together, our findings demonstrate that a subpopulation of phrenic motor neurons express *Npn-1* during initial phrenic nerve targeting towards the PFFs, as well as at later stages, when sophisticated innervation of the breathing muscle is established.

### Pre-diaphragm fasciculation of the phrenic nerves is only mildly disturbed upon systemic or motor-neuron-specific ablation of Sema3–Npn-1 signaling

**Fig. 2.**
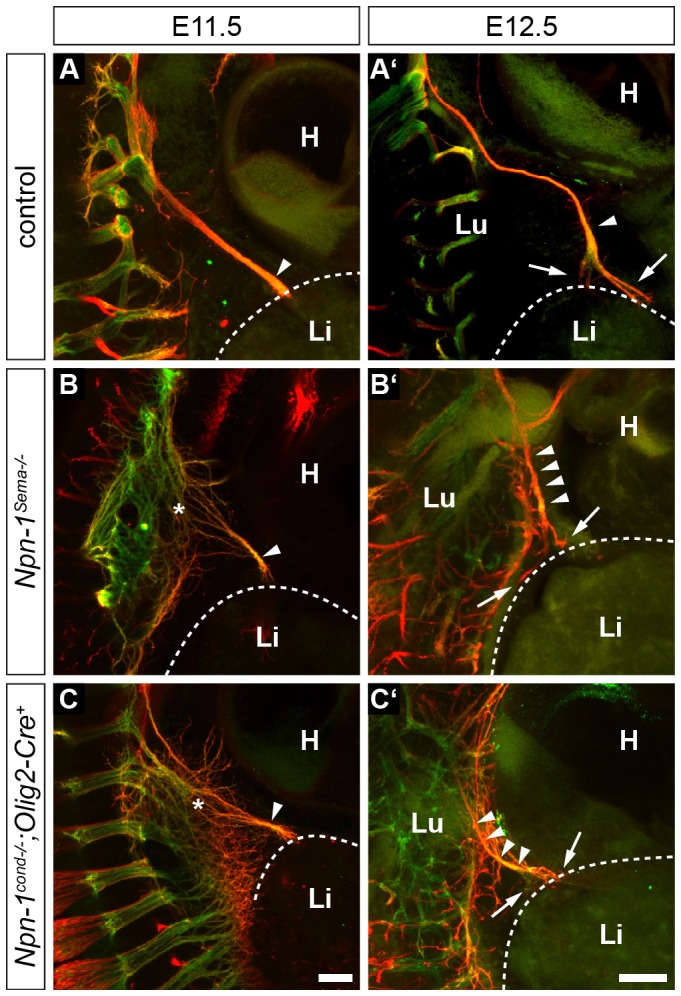
**PPFs are correctly targeted by phrenic nerves.** Whole-mount immunohistochemistry against eGFP-tagged Hb9 (somatic motor projections, green) and neurofilaments (motor and sensory axons, red) at stage E11.5 (A–C) and E12.5 (A′–C′). Control embryos show fasciculated phrenic projections after the brachial plexus at E11.5 (A, arrowhead). At the same stage, *Npn1^Sema−/−^* (B) and *Npn-1^cond−/−^;Olig2-Cre^+^* mutant embryos (C) expose severely disorganized projections within the brachial plexus region (asterisks). Phrenic axons reach the developing diaphragm as a fasciculated nerve branch regardless of the genotype (A–C, arrowheads). The phrenic nerve correctly targets the primordial developing diaphragm and starts to branch dorsally and ventrally at E12.5 (A′–C′, arrows). H, heart; L, liver; Lu, lung. Scale bars: 200 µm.

After verification of *Npn-1* expression in phrenic motor neurons during innervation of the diaphragm muscle, we investigated whether loss of the Sema3–Npn-1 signaling pathway affects phrenic axon targeting to the PPFs during early embryonic development. We performed immunohistochemistry on whole-mount preparations of E11.5 embryos, when the phrenic nerves project towards the PPFs (Fig. 2A), and at E12.5, when they have reached the developing diaphragm (Fig. 2A′) and are starting to branch and innervate newly formed myofibers (Merrell et al., 2015).

Upon systemic ablation of Sema3–Npn-1 signaling in *Npn-1^Sema−/−^* mice, we observed a severe defasciculation of spinal nerves within the brachial plexus, and slightly disorganized phrenic projections when leaving the plexus region at E11.5 (Fig. 2B, asterisk). However, phrenic axons fasciculated to one specific bundle right after leaving the plexus region (Fig. 2B, arrowhead). By E12.5, the phrenic nerves had reached the PPFs in a fasciculated manner and initially formed dorsal and ventral projections of the phrenic nerve that are comparable to those in wild-type littermates (Fig. 2B′, arrowheads, arrows).

To elucidate whether Npn-1 is responsible for fasciculation of the phrenic nerves in a cell autonomous manner, we selectively eliminated *Npn-1* from all somatic motor neurons in *Npn-1^cond−/−^;Olig2-Cre^+^* mutant embryos. Although axons were defasciculated within the plexus region, a distinct phrenic nerve projection that targets the PPF was also formed in these conditions (Fig. 2C, arrowhead, asterisk). During the initial branching within the costal muscle at E12.5, no obvious alterations were observed in *Npn-1^cond−/−^;Olig2-Cre^+^* mutant embryos when compared to control littermates (Fig. 2C, arrowheads).

Thus, although spinal nerves contributing to the brachial plexus are strongly defasciculated in *Npn-1^Sema−/−^* and *Npn-1^cond−/−^;Olig2-Cre*^+^ mutant embryos (Huber et al., 2005; Huettl et al., 2011), Sema3–Npn-1 signaling has no effect on initial specific branch formation and guidance of the phrenic nerves.

### Npn-1 cell autonomously governs phrenic nerve fasciculation within the diaphragm muscles

**Fig. 3.**
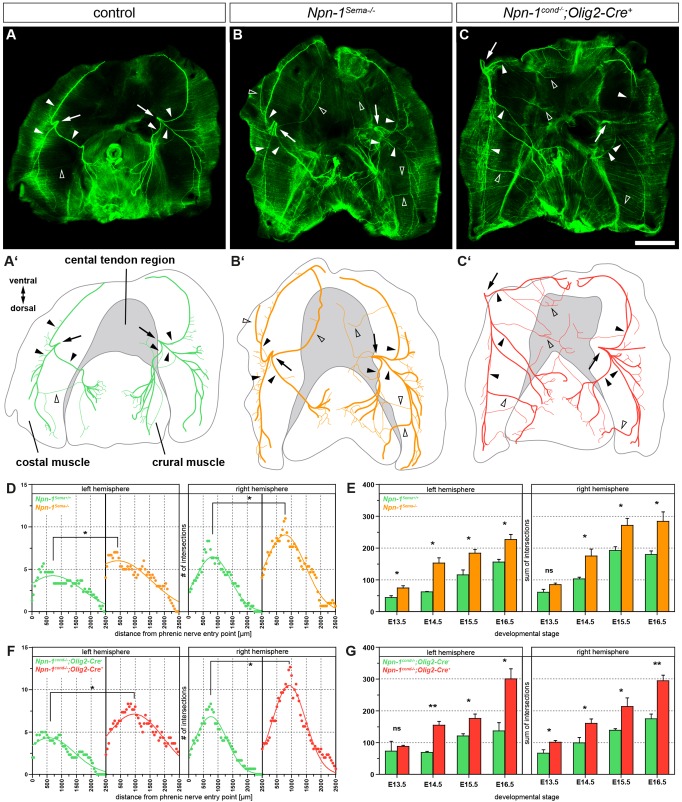
**Npn-1 cell autonomously governs phrenic nerve fasciculation within the diaphragm muscles.** (A–C) Whole-mount diaphragm staining against neurofilaments and synaptophysin (green). At E16.5, the phrenic nerves bilaterally form three distinct branches (filled arrowheads) after the phrenic nerve entry points (arrows) and show a tight innervation at the midline of costal myofibers (A,A′). *Npn-1^Sema−/−^* and *Npn-1^cond−/−^;Olig2-Cre^+^* mutant embryos reveal a severely defasciculated innervation pattern (B,B′,C,C′, open arrowheads). (A′–C′) Schematic representations of phrenic nerve growth within the diaphragm at developmental stage E16.5. Scale bar: 1 mm. (D–G) Branching of the right and left phrenic nerves within the costal muscles during development of the diaphragm was quantified by Sholl analysis centered at the phrenic nerve entry point. At E16.5 (D), the phrenic nerve of wild-type embryos branches significant less on both costal muscle hemispheres compared to their *Npn-1^Sema−/−^* mutant littermates (*n*=3, *P*≤0.05). Specific ablation of *Npn-1* from somatic motor neurons (F) reveals a similar significant higher branching of the phrenic nerve in mutant embryos compared to littermate controls (*n*=3, *P*≤0.05). Comparison of the sum of intersections between *Npn-1^Sema−/−^* (E) and *Npn-1^cond−/−^;Olig2-Cre^+^* (G) mutant embryos shows a significant difference from developmental stage E14.5 onwards in both hemispheres. Data represents mean±s.e.m. *P≤0.05; **P≤0.01; ns, not significant (Mann–Whitney U-test).

To investigate whether loss of Sema3–Npn-1 signaling affects phrenic nerve fasciculation and targeting during the innervation of the newly formed myofibers of the developing diaphragm, we stained whole diaphragms of E16.5 embryos with antibodies against neurofilaments and synaptophysin to label axons and nerve terminals, respectively. In control embryos, phrenic motor axons entered the diaphragm at the left and right hemisphere at the height of the vena cava (Fig. 3A,A′, arrows) and formed three distinct projections, one of which targeted the crural muscle, and two branches that innervated the ventral and dorsal proportion of the costal musculature (Fig. 3A,A′, arrowheads) with distinct branching patterns in each hemisphere. In addition, some thin axon bundles left their normal track in the costal muscle and projected towards the crural muscle of the diaphragm in wild-type embryos (Fig. 3A,A′, empty arrowheads). Systemic (Fig. 3B,B′, arrowheads) or motor-neuron-specific ablation of Sema3–Npn-1 signaling (Fig. 3C,C′, arrowheads) led to a severe defasciculation of phrenic motor projections within costal and crural musculature. To quantify the branching abnormalities within the costal muscles, we performed a Sholl analysis with concentric circles at the phrenic nerve entry points and counted axon intersections with each ring. At E16.5, when costal muscles are morphologically fully developed and innervated by phrenic nerve branches, nerve intersections with Sholl rings had significantly increased by ∼42% in the left and 40% in the right costal muscle hemisphere in *Npn-1^Sema−/−^* mutant embryos when compared to wild-type controls (Fig. 3D). Conditional ablation of *Npn-1* in phrenic motor neurons also led to a significant increase to 63% of aberrant costal nerve branching in the left hemisphere, and 54% in the right hemisphere of mutant diaphragms (Fig. 3F). We further analyzed costal diaphragm innervation in earlier developmental stages in *Npn-1^Sema−^* and *Npn-1^cond^;Olig2-Cre* mouse lines by adding up all phrenic nerve intersections with Sholl circles (sum of intersections). Although the sum of intersections was not consistently significantly different for both costal hemispheres at E13.5, phrenic nerve branching was significantly higher from E14.5 onwards, with an approximate increase of 78% in the left and 55% in the right hemisphere in both mutant mouse lines when compared to control littermates (Fig. 3E,G).

As correct centric formation and maintenance of NMJs is essential for normal muscular function, we sought to determine whether the significant extension of axon branching in mutant mice affects the generation of a tight NMJ band (Hippenmeyer et al., 2007; Tezuka et al., 2014). Even though we did observe a severe defasciculation of phrenic axons in the diaphragm, quantification of the NMJ band width in *Npn-1^Sema−/−^* and *Npn-1^cond−/−^;Olig2-Cre*^+^ mutants showed that there was no significant difference when compared to wild-type embryos at E16.5 (Fig. S2A–D).

Our results thus show that Sema3–Npn-1 signaling cell autonomously governs phrenic nerve fasciculation and branching during innervation of the costal muscles of the diaphragm, whereas NMJ patterning is not altered by defective fasciculation of phrenic axons.

### Misprojecting phrenic axons innervate ectopic muscles in the central tendon region of the diaphragm

**Fig. 4.**
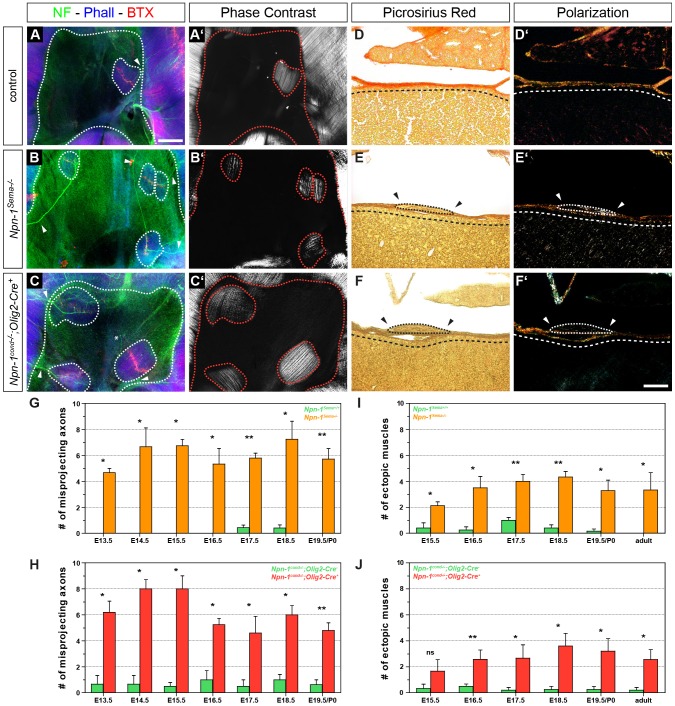
**Phrenic axons misproject into the central tendon region and innervate ectopic muscles in both mutant embryos.** Whole-mount diaphragm staining against neurofilaments and synaptophysin (green), actin (blue) and neuromuscular junctions (NMJs, red). At E16.5, the majority of misprojecting axons innervate ectopic muscles in the central tendon region of mutant embryos (B,C, dotted lines), and very scarcely also in wild-type embryos (A). Rare exceptions where an aberrantly projecting axon is not targeting ectopic musculature were observed (C, asterix). Phase contrast microscopy displays typically aligned myofiber orientation that is comparable to native costal muscles of the diaphragm (A′–C′). Picrosirius Red staining on frontal sections of E16.5 *Npn-1^Sema−/−^* (E) and *Npn-1^cond−/−^;Olig2-Cre^+^* (F) mutant embryos shows that ectopic muscles (dotted lines) are lying on top of the central tendon region (dashed lines), whereas the majority of wild-type animals show an ectopic muscle-free central tendon region (D). Polarization microscopy further underpin the results, as they stretch on top of birefringent collagen fibers of the central tendon region (D′–F′). Arrowheads indicate the lack of collagenous extracellular matrix around ectopic muscles (E–F′). Scale bars: 200 µm (A–C′), 100 µm (D–F′). Quantification reveals significantly more axons that misproject into the central tendon region in *Npn-1^Sema−/−^* (G) and *Npn-1^cond−/−^;Olig2-Cre^+^* (H) mutant embryos when compared to control embryos during the phase of diaphragm development. Interestingly, quantification of ectopic muscles in both mutant mouse lines reveals a slight, but not significant, increase of ectopic muscles until the beginning of muscle hypertrophy at E16.5, which then slightly decreased until adulthood (I,J). Data are represented as mean±s.e.m. (*n*≥3). *P≤0.05; **P≤0.01; ns, not significant (Mann–Whitney U-test).

The central tendon region of the diaphragm consists of tendinous tissue that provides functional force transduction between both costal muscle hemispheres. Normally, this region is free of muscles and/or nerve endings, and only harbors vessels in close proximity to the medial part of the costal muscles (Stuelsatz et al., 2012). Remarkably, we observed a significant 8.7-fold increase in misprojecting axon numbers into the central tendon region of *Npn-1^Sema−/−^* mutants when compared to control embryos (empty arrowheads on Fig. 3B′, and arrowheads on Fig. 4A,B) at E16.5. This misinnervation had already started during initial innervation of the diaphragm at E13.5 (Fig. 4G). During development of the diaphragm, misprojected axons remained elevated in *Npn-1^Sema−/−^* mutants; however, the number of misguided axons did not change significantly over developmental stages (Fig. 4G). Intriguingly, upon closer investigation of the central tendon region, we observed ectopically formed muscle patches in embryos where Sema3–Npn-1 signaling was systemically abolished, which were evident from E15.5 until late adulthood. The number of ectopic muscles was significantly higher in *Npn-1^Sema−/−^* mutant embryos with a mean increase of 9.5-fold compared to wild-type embryos (Fig. 4I), and showed a phenotype of typically aligned myofibers, comparable to native costal muscles, whereas the orientation of these muscle patches did not correlate with costal muscle fibers (Fig. 4A′,B′; Fig. S3A–A″).

As *Npn-1* is also expressed in muscle progenitors of the diaphragm (see Fig. 7G), we sought to determine the underlying effect of Sema3–Npn-1 signaling on ectopic muscle formation. Therefore, we utilized conditional ablation of *Npn-1* in all somatic motor neurons, including phrenic motor neurons, to analyze whether mis-migration of muscle progenitors into the central tendon region was caused by systemic loss of Sema3–Npn-1 signaling. *Npn-1^cond−/−^;Olig2-Cre^+^* mutant embryos showed a similar pattern of misprojecting axons towards the central tendon region when compared to *Npn-1^Sema−/−^* mutants, with no clear favor of a specific part of the central tendon region (Fig. 4C) and, intriguingly, also showed innervated ectopic muscles with a striated muscle fiber pattern (Fig. 4C,C′). Quantification showed a significant average increase of 9.3-fold for the number of axons misprojecting into the central tendon region (Fig. 4H) and a 11.6-fold increase for ectopic muscles (Fig. 4J) in *Npn-1^cond−/−^;Olig2-Cre^+^* mutant embryos when compared to *Cre*^−^ control embryos. Misprojecting axons and ectopic muscles did not change significantly during later development in *Npn-1^cond−/−^;Olig2-Cre^+^* animals. Interestingly, nearly all of these ectopic muscles were innervated by misprojected axons (97.66±1.05%, mean±s.e.m.), whereas approximately one tenth (12.25±2.56%) of the misprojected axons did not innervate ectopic muscles. Additionally, orientation of ectopic muscles in relation to costal muscle fibers revealed that the majority ectopic muscles adapt approximately an 45° angle in comparison to costal muscle fibers (Fig. S3A–A″). Furthermore, ectopic muscle size within both mutant mouse lines was not significantly different when compared to the very rarely observed ectopic muscles in wild-type animals at all developmental stages (Fig. S3B,C).

To assess the underlying reason for ectopic muscle formation, we investigated the localization of ectopic muscles within the central tendon region of the diaphragm. On the one hand, transdifferentiation of PPF fibroblasts, which migrate on the septum transversum and later form the central tendon region (Merrell et al., 2015), might cause ectopic muscles that are embedded within the central tendon region. On the other hand, migrating myoblasts might form ectopic muscles, which would lie directly on top of the central tendon region. Indeed, Picrosirius Red staining on frontal paraffin sections of mutant diaphragms at E16.5 indicates that newly formed muscles are located on top of the central tendon region (Fig. 4E,F, arrowheads and dashed line, respectively), thus arguing for the latter. Polarization light microscopy on Picrosirius-Red-stained diaphragm sections furthermore revealed thick collagen I fibers below ectopic muscles (Fig. 4E′,F′; yellow–orange birefringence), which mark the highly organized collagenous central tendon region (Fig. 4D,D′).

Taken together, phrenic axons misproject at a significantly higher rate into the central tendon region of the diaphragm and innervate ectopic muscles in both mutant mouse lines. These ectopic muscles appear to derive from muscle progenitors that are mismigrating on top of tendinous tissue of the central tendon region. Given the fact, that ectopic muscles are also established upon motor-neuron-specific elimination of *Npn-1*, a direct effect of Sema3–Npn-1 signaling on migrating myoblasts appears unlikely and thus secondary effects of axons mismigrating into the central tendon region might cause ectopic muscle formation.

### Slit2 and Robo1 are expressed in phrenic motor neurons and myoblasts forming costal muscles, respectively, during diaphragm development

**Fig. 5.**
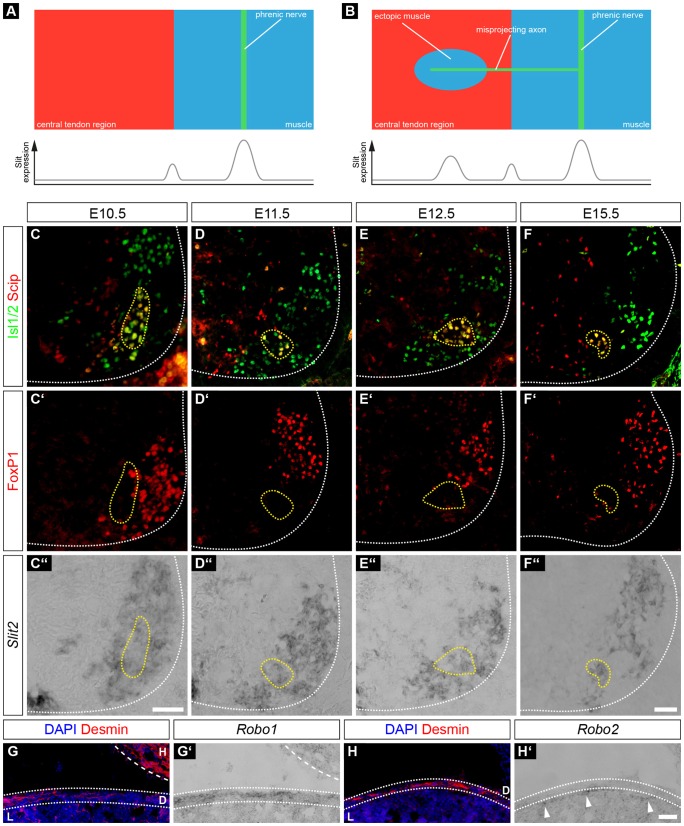
**Slit2 and Robo1 are expressed in phrenic motor neurons and muscle progenitors, respectively, during diaphragm innervation.** (A,B) Schematic view of hypothetical phrenic nerve and myoblast interaction during diaphragm development. In wild-type embryos, Slit is released from cells of the PPF at the border between central tendon region and premature muscle. In addition, Slit is also accumulated in the proximity of phrenic growth cones at the midline of newly formed costal muscles where it attracts resident muscle progenitors (A). In *Npn-1^Sema−/−^* and *Npn-1^cond−/−^;Olig2-Cre^+^* mutant animals, axons that misproject into the central tendon region release Slit and attract some resident muscle progenitors, which later fuse to striated myofibers (B). Slit2 is strongly expressed in lateral motor column motor neurons (FoxP1^+^ cells, C′–F′,C″–F″) and a subpopulation of phrenic motor neurons (Scip^+^, Isl1^+^ and FoxP1^−^) between E10.5 and E15.5 (C,D,C″–F″). Although Robo1 (G′) is expressed within the desmin^+^ cells of the costal muscles (G), Robo2 (H′) is expressed by cells of the intermediate zone between the diaphragm and the liver (H, H′, arrow heads). H, heart; D, diaphragm; L, liver. Scale bars: 200 µm.

Recent findings have revealed that Sli–Robo signaling does not only have a direct effect on phrenic nerve fasciculation (Jaworski and Tessier-Lavigne, 2012), but that Slit proteins, released by the sclerotome, can also repel pioneering myoblasts during early development of avian embryos (Halperin-Barlev and Kalcheim, 2011). At later stages, migrating pioneering myoblasts are attracted by Slit proteins released by the tendon to guide them to their final destination in *Drosophila* (Kramer, 2001). We hypothesized that Slit–Robo signaling between motor neuron growth cones and pioneering and/or migrating myoblasts might be the underlying origin of ectopic muscle formation in *Npn-1^Sema−/−^* and *Npn-1^cond−/−^;Olig2-Cre^+^* mutant mice. In wild-type animals, migrating myoblasts might be attracted by Slit that is released from the myotendinous junction between the central tendon region and costal muscles. In addition, Slit, secreted by motor neuron growth cones during muscle innervation concentrated at the NMJ band (Fig. 5A) and might act as a migration target for secondary myogenesis. Accordingly, *Olig2* mutants, in which all somatic motor neurons and their projections are absent, showed a significantly thinned diaphragm muscle (data not shown). In contrast to that, phrenic axons misprojecting into the central tendon region in embryos with systemic or motor-neuron-specific ablation of Sema3–Npn-1 signaling were still able to release Slit proteins, which subsequently might pull some myoblasts out of the developing costal muscles as axons aberrantly invade the tendon region. Within this newly formed microenvironment composed of motor neuron growth cones and myoblasts, myoblast fusion due to autocrine signaling between neighboring myoblasts and/or paracrine trophic support, provided by the ectopic growth cone, might contribute to ectopic muscle formation (Fig. 5B).

**Fig. 6.**
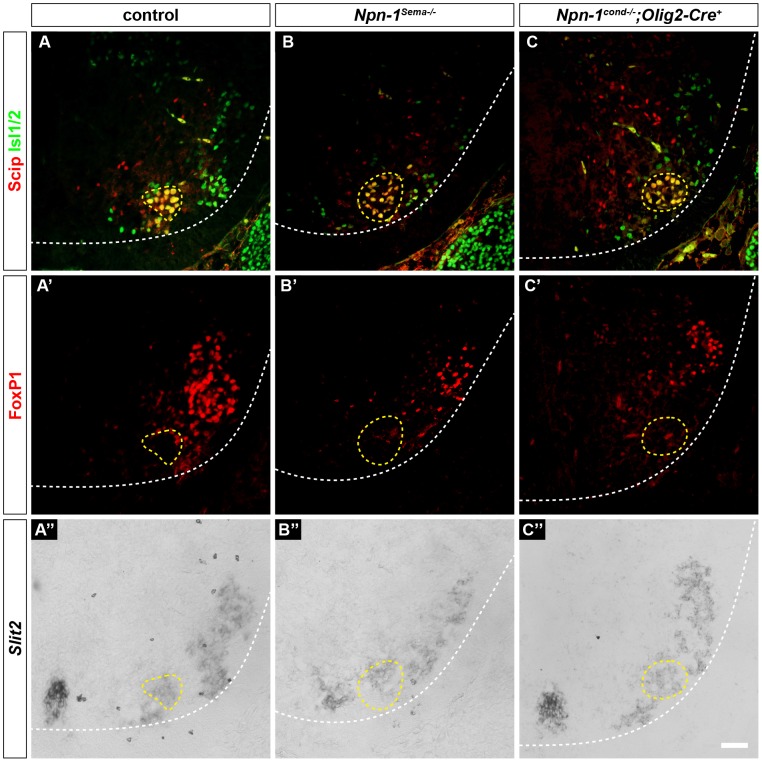
**Slit2 expression is not changed upon manipulation of Sema3–Npn-1 signaling.** Phrenic motor neurons express Scip (red) and Isl1 or Isl2 (green) and are negative for FoxP1 (red) (A–C,A′–C′). Slit2 expression in phrenic motor neurons (yellow dotted lines) is unchanged in *Npn-1^Sema−/−^* (B–B″) and *Npn-1^cond−/−^;Olig2-Cre^+^* (C–C″) mutant animals when compared to control embryos (A–A″) at developmental stage E16.5. Scale bar: 200 µm.

**Fig. 7.**
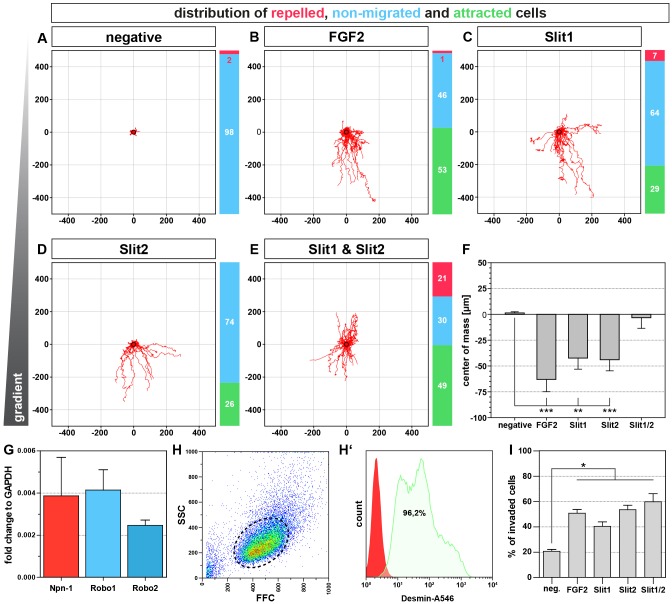
**Slit1 and Slit2 can attract primary muscle progenitors.** Chemotaxis assays with primary costal muscle progenitors of the diaphragm in a collagen I matrix reveal that ∼50% of all cells are attracted by FGF2 (B) whereas cells are hardly attracted without any chemokine (A). In contrast, Slit1 (C) and Slit2 (D) attract almost one third of muscle progenitors. Exposure of muscle progenitors to a combination of Slit1 and Slit2 further enhances the attractive effect, although roughly one fifth of the cells turn towards repulsion (E). Quantification of migrating cell direction by determining the center of mass shows that FGF2, as well as Slit1 and Slit2 significantly attracted muscle progenitors, whereas the center of mass did not change with the combination of both Slit proteins. Quantitative PCR on E14.5 costal diaphragm muscles validates the expression *Robo1* and *Robo2* during muscle progenitor migration and fusion and reveals that *Npn-1* is also upregulated in cells within the developing diaphragm muscle (G). Isolated primary muscle progenitors have a relatively homogenous size (H) and the majority of primary muscle progenitors are positive for the myogenic marker Desmin (H′). Transwell migration assay shows that Slit1 and Slit2, as well as the combination of both are strongly attractive when compared to the negative control without any ligand addition. FGF2, a known muscle progenitor attractant served as a positive control (I). Quantitative results are mean±s.e.m. (*n*=4 independent experiments). *P≤0.05, **P≤0.01, ***P≤0.001 (Mann–Whitney U-test). Axis scaling for A–E is in µm.

To analyze whether Slit and Robo are expressed during the phase of diaphragm muscle development and axon guidance, we performed *in situ* hybridization on spinal cord sections and the costal muscle at crucial developmental time points. During diaphragm innervation, *Slit2* (Fig. 5C″–F″) was strongly expressed in the majority of Scip^+^ and Isl1^+^ motor neurons in the PMC between E10.5 and E12.5, and at E15.5 (Fig. 5C–F). Slit1 was strongly expressed in the floor plate, but showed no noticeable expression in the ventral horn (data not shown). Correspondingly, mRNA encoding the Slit receptor Robo1 (Fig. 5G′) was expressed in Desmin^+^ muscle progenitors (Fig. 5G) at the developmental stage E15.5, when muscle progenitors migrate and fuse on the septum transversum. In contrast, *Robo2* (Fig. 5H′) was weakly expressed in cells within the intermediate zone between the diaphragm musculature and the liver (Fig. 5H). Expression of Robo1 and Robo2 in costal diaphragm cells was further validated by quantitative PCR (qPCR) on whole costal diaphragm RNA isolation at E14.5 (see Fig. 7G). Furthermore, *Slit2* expression of motor neurons in the PMC was not changed in *Npn-1^Sema−/−^* and *Npn-1^cond−/−^;Olig2-Cre*^+^ mutants when compared to control embryos (Fig. 6).

Thus, Slit and Robo mRNAs are present in phrenic motor neurons and myoblasts, respectively, at the time when ectopic muscles are generated, and might contribute to ectopic muscle formation in the central tendon region upon misguidance of phrenic axons into the central tendon region.

### A subpopulation of primary muscle progenitors is attracted by Slit1 and Slit2

To analyze the functional effect of Slit ligands on muscle progenitors, we carried out chemotaxis experiments in a 3D collagen I matrix. The majority of isolated costal diaphragm muscle progenitors of E14.5 embryos were positive for the muscle-specific marker desmin (Fig. 7H,H′). Primary muscle progenitors that were not exposed to a chemotaxis gradient did not show a high migration potential (Fig. 6A), whereas fibroblast growth factor 2 (FGF2), a well-known attractant for myogenic cell lines (Bischoff, 1997; Corti et al., 2001), attracted 53% of all cells, although 46% of the cells did not show a repulsive or attractive response (Fig. 7B). Treatment with either Slit1 (Fig. 7C) or Slit2 (Fig. 7D) attracted 29% and 26% of cells, respectively, when compared to untreated cells. The combination of Slit1 and Slit2 further raised the proportion of attracted primary cells to 49%. Interestingly, a higher fraction of primary muscle progenitors are repelled by the combination of Slit1 and Slit2 (21%) when compared to Slit1 (7%) or Slit2 (0%) alone (Fig. 7C–E). In addition, we calculated the center of mass of all cells after 24 h to quantify the direction of migration (Fig. 7F). Whereas cells that had not been exposed to a gradient, showed a non-directed migration, FGF2 treatment strongly attracted muscle progenitors. Slit1 or Slit2 copied the attractive effect on migration of muscle progenitors to a lesser extent, whereas combined exposure of Slit1 and Slit2 led to a non-directed migration for the whole cell population comparable to the negative control. To further verify an attractive effect of Slit proteins on muscle progenitors, we used a transwell invasion setup. Approximately 20% of all cells migrated towards the non-seeded side when no ligand was added. In contrast, exposure of primary cells to FGF2 caused a significant increase to ∼50% of migrating cells. Likewise, Slit1 and Slit2 caused a significant increase to 40% and 55%, respectively, when compared to untreated cells. Additionally, muscle progenitors suspended with a combination of Slit1 and Slit2 showed a similar effect of increased migration, which is comparable to the 3D chemotaxis results (Fig. 7I).

Taken together, a subpopulation of primary cells from costal diaphragm muscles at E14.5 are attracted by Slit1 and Slit2, and the combination of both ligands results in a cumulative attractive effect, whereas some primary progenitors turned towards repulsion, leading to an overall effect of non-directed migration within the population.

## DISCUSSION

Development and maturation of neuronal circuits are pivotal for functional innervation of muscles and thereby the viability of any organism. Understanding the complete genetic fundament of phrenic nerve and diaphragm development is thus crucial to establish treatments for developmental or postnatal degenerative respiratory diseases. Therefore, we employed two different genetic approaches to uncover the influence of Sema3–Npn-1 signaling on phrenic nerve targeting of the PPF and branching within the diaphragm. Intriguingly, phrenic axons not only misprojected into the central tendon region, but also innervated ectopically generated musculature, thus raising questions concerning the primary mechanisms that trigger (ectopic) muscle formation.

### Sema3–Npn-1 signaling is not involved in early targeting of the PPF, but affects the later innervation of the diaphragm

Prevention of signaling between Sema3A and its receptor Npn-1 leads to premature ingrowth, defasciculation of limb innervating sensory and motor projections, and axon pathfinding defects during the development of neuronal circuits (Huber et al., 2005; Huettl et al., 2011). We show here that motor neurons of the PMC express *Npn-1* during axon targeting towards the brachial plexus, their stopover at the PPFs and the final branching within the developing diaphragm. Systemic or motor-neuron-specific elimination of Sema3–Npn-1 signaling causes severe defasciculation of spinal nerves, including designated phrenic axons, within the brachial plexus region; however, it does not affect fasciculation of the phrenic nerves to one distinct branch after leaving the plexus region or targeted growth towards the PPF. Thus, phrenic axons are not affected like axons originating from the lateral motor column that innervate limb musculature (Huber et al., 2005; Huettl et al., 2011). This indicates the utilization of different guidance mechanisms for limb-innervating and phrenic axons in the brachial plexus to avoid a black-out or an intermixture of axon tracts of both respiratory and locomotor systems upon loss of one peripheral axon guidance system. Interestingly, systemic deletion of the homeobox transcription factor Hb9 (also known as MNX1), leads to a severe misprojection of various classes of motor axons, including the phrenic nerves. In *Hb9*-knockout embryos, phrenic axons are either redirected into the limb bud, or phrenic motor neurons are not specified at all (Thaler et al., 1999). Similar phrenic nerve guidance defects into the limb or epiaxial mesenchyme in *Npn-1^Sema−/−^* and *Npn-1^cond−/−^;Olig2-Cre*^+^ mutants cannot be completely excluded, and might be uncovered by the combination of retrograde tracings of limb or epaxial projecting axons and specific neuronal markers of the PMC. One mechanism that might govern correct early fasciculation of the phrenic nerves is the interaction of the transmembrane glycoprotein activated leukocyte cell adhesion molecule (ALCAM) with its membrane-bound binding partner CD6, as it is specifically expressed at brachial levels in motor neurons of the PMC or all somatic motor neurons, respectively, and has been shown to be involved in axon fasciculation (Philippidou et al., 2012; Pourquié et al., 1990; Weiner et al., 2004).

In contrast to the moderate phenotype during early phrenic nerve targeting, the *Npn-1^Sema−/−^* and *Npn-1^cond−/−^;Olig2-Cre^+^* mutant animals reveal a severe increase of axon branching over the whole period of diaphragm development. Interestingly, both mouse lines are born at normal Mendelian ratios, however, only ∼70% of *Npn-1^cond−/−^;Olig2-Cre^+^* and 60% of *Npn-1^Sema−/−^* mutant embryos survive the first postnatal week (Gu et al., 2003), providing evidence for a neonatal and not a developmental effect. The substantial incidence of neonatal lethality might indicate that the degree of defasciculation and therewith myofiber innervation might have an effect on neonatal survival. For example, in animals where *Hox5* and *Unc5c* were mutated, a profound reduction of costal muscle innervation of the diaphragm, which leads to neonatal cyanosis, is observed (Burgess et al., 2006; Philippidou et al., 2012). Furthermore, systemic ablation of *erbB2* results in a similar defasciculation of the phrenic nerves during innervation of both diaphragm muscles. In contrast to *Npn-1^Sema−/−^* and *Npn-1^cond−/−^;Olig2-Cre^+^* mutant embryos, deletion of *erbB2* leads to a degeneration of NMJs from E14.5 onwards, due to the complete loss of NMJ-stabilizing Schwann cells, and thus results in neonatal death (Lin et al., 2000; Morris et al., 1999). Intriguingly, conditional gain-of-function experiments with early (Myo-Cre^+^) or later (HSA-Cre^+^) muscle-specific stabilization of β-catenin shows a likewise strong increase of phrenic nerve branching that is related to a broadening of the centered NMJ band (Liu et al., 2012; Wu et al., 2012).

Thus, Sema3–Npn-1 signaling acts cell autonomously during diaphragm innervation on phrenic nerve axon branching throughout diaphragm development whereas NMJ formation is normal. However, whether the existence of ectopic musculature in the central tendon region contributes to neonatal lethality of both mouse lines used in our study has to be further investigated.

### Ectopic muscles in the central tendon region of the diaphragm are innervated by misrouted axons of the phrenic nerves after ablation of Sema3–Npn-1 signaling

Interestingly, next to the defasciculation of the phrenic nerves in both mouse lines, we observed axons that occasionally projected aberrantly into the central tendon region, and the majority of these misguided axons innervated ectopic muscles. These patches were maintained until late adulthood and thus imply a functional innervation by the phrenic nerves. These findings raise the question whether misprojected axons or misplaced muscle progenitors initiate the process of ectopic muscle formation. The development of ectopic muscles might have different causes. First, this might be caused by transdifferentiation of tendon progenitor cells or fibroblasts of the PPF to muscle progenitors or directly into myoblasts owing to yet unknown secreted cues of the misprojected growth cones. Second, another possible reason is the attraction and/or condensation of migrating myoblasts towards the growth cones of misguided phrenic axons and the following cellular fusion.

The current opinion of diaphragm muscle development consists of pioneering myoblasts that delaminate from the ventrolateral lip of the dermomyotome and migrate to their final mesenchymal compartment to provide a target point for following mitotically active myoblasts that then will fuse to myofibers (Babiuk et al., 2003; Dietrich et al., 1999; Merrell et al., 2015). We favor a migratory mechanism of muscle progenitors due to the position of ectopic muscles on top of the central tendon region. Although it might be possible that mis-migration of muscle progenitors is caused by the lack of Sema3–Npn-1 signaling in *Npn-1^Sema−/−^* mutant embryos, the fact that tissue-specific ablation of *Npn-1* receptors in phrenic motor neurons mimics both aberrant projections into the central tendon region and ectopic muscle formation renders the direct involvement of this pathway in muscle progenitor migration unlikely. Therefore, a secondary mechanism that is independent of Sema3–Npn-1 signaling might underlie the mis-migration of muscle progenitors into the central tendon region and subsequent myocyte fusion. This signaling could be mediated by secreted ligands or by direct interaction of growth cones and muscle progenitors during innervation of developing (ectopic) muscles.

One possibility for ligands secreted by phrenic growth cones would be Sema3A, as *in vitro* experiments with mouse myoblast cell lines indicate that Sema3A can upregulate myogenin (Suzuki et al., 2013), an indispensable transcription factor during skeletal muscle development (Nabeshima et al., 1993). Sema3A is also expressed in the PMC (data not shown), and its secretion from misprojecting growth cones into the central tendon region might induce myogenesis in PPF fibroblasts within the central tendon region by the induction of a similar myogenic pathway. Contrary to this idea, chemotaxis assays with recombinant Sema3A revealed a strongly repulsive effect on muscle progenitors (data not shown) and therefore condensation of muscle progenitors around misprojected growth cones is implausible.

Contact-mediated fusion of myocytes that is initialized by the interaction with motor neuron growth cones is another possibility. Even if a direct ligand–receptor system that stimulates myocyte fusion is currently unknown, conditional stabilization of β-catenin in skeletal muscle cells results not only in an increased branching of the phrenic nerves along the costal muscles of the diaphragm, but also in the establishment of innervated ectopic muscles in the central tendon region that form NMJ bands (Wu et al., 2012). The authors postulate that newly formed ectopic muscles attract misprojecting axons during development of the diaphragm from E14.5 onwards. In contrast, we observed misprojecting axons as early as E13.5, before fused myofibers were visible at E15.5. This discrepancy illustrates that the sequence of muscle development and innervation has to be further elucidated.

In conclusion, our results demonstrate that a cell autonomous effect of Sema3–Npn-1 signaling governs phrenic nerve fasciculation during diaphragm muscle innervation, whereas a direct involvement of *Npn-1* in ectopic muscle formation is rather unlikely. Nevertheless, the specific function of Sema3–Npn-1 signaling during muscle development is currently unknown.

### Diaphragm myoblasts from costal muscles are attracted by Slits

As *Olig2* is not expressed in cells of the diaphragm (Fig. S4) and thereby Sema3–Npn-1 signaling is not affected in muscle progenitors, we hypothesized a secondary underlying mechanism that causes misguided myoblast migration and subsequent fusion. The tight interaction of migrating phrenic growth cones and myoblasts provides a tightly specified micro-environment and therefore can also influence myoblast migration. The delamination and migration of muscle progenitors from the dermomyotome to their final targets is crucial for the correct patterning of the musculoskeletal system. Disturbance of native guidance signals, such as hepatocyte growth factor to c-Met signaling, or ablation of its upstream transcription factors like Pax3 or Lbx1, leads to severely impaired skeletal muscle phenotypes (Brohmann et al., 2000; Dietrich et al., 1999; Swartz et al., 2001), and therewith also perturb diaphragms formation (Babiuk and Greer, 2002; Babiuk et al., 2003).

We provide a first indication that Slit1 and Slit2 have an attractive effect on *in vitro* cultured muscle progenitors of the developing costal diaphragm muscles. Besides our postulated mechanism of muscle progenitor guidance by motor neuron growth cones *in vivo*, axonal-derived Slit2 could also be involved in NMJ stabilization, as muscle-intrinsic Slit2 can compensate NMJ formation when β-catenin is deleted in a skeletal actin background (Wu et al., 2015). Therefore, Slit2 might be essential for anterograde as well as retrograde signaling between axonal tips and myocytes.

Interestingly, some primary cells turn towards repulsion when Slit1 and Slit2 proteins are combined, which possibly relies on receptor complex formation between Robo and their co-receptors and thereby modification of downstream signaling (reviewed in Ypsilanti et al., 2010). Nevertheless, it is unlikely that axon-derived Slit1 has a major role *in vivo*, as it is not expressed in phrenic motor neurons during the innervation of the diaphragm (Jaworski and Tessier-Lavigne, 2012).

In *Drosophila* embryos, Slit that is released from the midline and is involved in early repulsion of muscle progenitors (Kramer, 2001). Furthermore, it seems that Slit-mediated repulsion during delamination of muscle progenitors from the dermomyotome is phylogenetically conserved, as Slit1 directs the migration in a somite-intrinsic manner in avian embryos (Halperin-Barlev and Kalcheim, 2011). Remarkably, initial Slit-mediated repulsion of muscle progenitors turns towards attraction at future muscle attachment sites. The main source of Slit during myocyte attraction in later development seems to be cells in these sites, e.g. tendon progenitor cells (Kramer, 2001).

Therefore, Robo^+^ muscle progenitors might be initially repulsed from the dermomyotome by sclerotome-released Slit1 and subsequently captured by Slit2 releasing motor neuron growth cones that project towards their final targets. It is unlikely that axonal Slit affects guidance of muscle progenitors during the primary myogenic wave, as ablation of all motor neurons by deletion of its Olig2^+^ progenitors does not affect overall diaphragm muscle formation, even though costal diaphragm musculature is weakened, thus arguing for an axonal involvement in muscle hypertrophy. Remarkably, muscle formation was altogether normal when Robo1 and Robo2, or Slit2 were systemically ablated, whereas phrenic axons are severely defasciculated (Jaworski and Tessier-Lavigne, 2012). Nonetheless, no axons misprojected into the central tendon region and ectopic muscle formation occurred in an extremely low incidence (personal communication with Alexander Jaworski, Brown University, Providence, RI). Thus, muscle progenitors might be especially sensitive to extracellular Slit signals during the secondary wave of myogenesis and misguided phrenic axons are very likely the underlying reason for ectopic muscle formation. However, only Slit2 ablation in motor neurons or deletion of Robo1 and Robo2 in muscle progenitors in combination with axon guidance disturbance, can elucidate the underlying mechanisms of Slit–Robo signaling *in vivo* in this system.

## MATERIALS AND METHODS

### Ethics statement

Animals were handled and housed according to the federal and institutional guidelines for the care and use of laboratory animals, approved by the Helmholtz Zentrum München Institutional Animal Care and Use Committee, and the government of Upper Bavaria.

### Mouse embryo preparation

Genotypes of mouse embryos were determined as described previously for *Npn-1^Sema−^* and *Npn-1^c^*^ond^ (Gu et al., 2003), *Olig2-Cre* (Dessaud et al., 2007) and *Hb9::eGFP* (Wichterle et al., 2002). *Npn-1^cond−/−^;Olig2-Cre^+^* mutant embryos were compared to littermate controls (*Npn-1^cond−/−^;Olig2-Cre^−^*) in all experiments with motor-neuron-specific ablation of *Npn-1*.

### Immunohistochemistry

The following primary antibodies were used: goat anti-FoxP1 (1:250, AF4534, R&D Systems), goat anti-Scip (1:250, sc-11661, Santa Cruz Biotechnology), mouse anti-synaptophysin (1:200, S5768, Sigma-Aldrich), mouse anti-Neurofilament 2H3 and mouse anti-Isl1 39.4D5 (1:50, obtained from the Developmental Studies Hybridoma Bank developed under the auspices of the NICHD and maintained by The University of Iowa, Department of Biological Sciences, Iowa City, IA 52242), rabbit anti-desmin (1:250, ab15200, Abcam) and rabbit anti-GFP (1:1000, A-11122, Invitrogen). Antibodies were visualized using fluorochrome-conjugated secondary antibodies (1:250, Jackson Dianova).

The protocols for whole-mount embryo staining and immunohistochemistry have been described previously (Huber et al., 2005; Huettl et al., 2011). For whole-mount diaphragm staining, mouse diaphragms were dissected from pre-fixed embryos, rinsed in PBS, incubated for 15 min in 0.1 M glycine in PBS and blocked overnight in blocking solution (3% BSA, 0.5% Triton X-100, 5% horse serum in PBS). Afterwards, diaphragms were incubated with primary antibodies in blocking solution overnight at room temperature. Diaphragms were washed three times for 30 min in washing buffer (0.5% Triton X-100 in PBS), blocked for 1 h in blocking solution and subsequently incubated with secondary antibodies in blocking solution over night at room temperature. Specimens were incubated with Alexa-Fluor-647-conjugated phalloidin (1:75, Life Technologies) and Rhodamin-coupled α-bungarotoxin (1:100, Life Technologies) in blocking solution for 1 h at room temperature, rinsed three times for 30 min in PBS and mounted with Mowiol. Images were obtained with a Zeiss LSM 510 or a Zeiss AxioObserver.

### Quantification of phrenic nerve defasciculation, misprojecting axons and ectopic muscles

To assess phrenic nerve defasciculation within the diaphragm, a Sholl analysis (Sholl, 1953) centered at the nerve entry point to the diaphragm was performed. Intersections of phrenic axons with concentrical circles every 50 μm were counted and afterwards fitted to a Gaussian regression curve. Statistics were calculated for the Gaussian curve amplitude. Ectopic muscle quantity and size were evaluated in phase-contrast images from developmental stage E15.5 onwards.

### Histological analysis

Embryos were fixed in 4% PFA in PBS and embedded in paraffin after dehydration. Frontal sections (8 μm) were deparaffinized, rehydrated and incubated for 1 h in 0.1% Direct-Red-80 (Sigma-Aldrich) in saturated aqueous picric acid staining solution. Sections were washed in 0.005% acidic acid and dehydrated before mounting. Sections were imaged by bright field and polarization microscopy. Ectopic muscle location within the central tendon region was determined by collagen I distribution as organized collagen fibers show red birefringence under polarized light, whereas muscle tissue appears yellow–green (Laws, 2004).

### *In situ* hybridization

*In situ* hybridization was carried out as described previously (Huber et al., 2005) using digoxigenin-labeled mouse riboprobes against *Npn-1* (Huettl et al., 2011), *Slit1*, *Slit2*, *Robo1* and *Robo2* (kindly provided by Nilima Prakash, Prakash et al., 2009). In brief, dissected embryos were fixed for 1 h in 4% PFA in PBS, cryoprotected in 30% sucrose in PBS and sectioned at 12-μm thickness on a cryostat. Sections were incubated with antisense probes, stringency washed and incubated overnight with anti-digoxigenin-AP FAB fragments (1:2000, Roche) in blocking buffer. The color reaction was carried out with NBT/BCIP (Roche) in 5% polyvinyl alcohol in reaction buffer. The reaction was stopped by washing with distilled water and subsequent immunohistochemistry was performed.

### Quantitative PCR

Diaphragms of E13.5 embryos were dissected and costal muscles were cleaned from crural muscles and the septum transversum. Tissue was minced, homogenized with a QiaShredder and total RNA was isolated using the RNeasy Micro Kit (Qiagen). RNA was reverse-transcribed using the first-strand cDNA synthesis kit (Roche) and quantitative PCRs were performed on a LightCycler 96 with a fast-start essential DNA green master mix (both Roche). Samples were normalized to the house-keeping gene *Gapdh*. The following primers were used: *Npn-1* (Mm.PT.56a.30361019), *Robo1* (Mm.PT.56a.32204547), *Robo2* (Mm.PT.56a.29354525), *Olig2* (Mm.PT.58.42319010) and *Gapdh* (Mm.PT.39a.1; Integrated DNA Technology).

### Myoblast chemotaxis assay

Diaphragms of eight to ten E14.5 embryos were dissected, and costal muscles were cleaned from crural muscles and the septum transversum. Muscle tissue was pooled, minced and incubated under continuous agitation in 250 U/ml collagenase II (Worthington) in DMEM (Life Technologies) for three times for 10 min each with a final trituration. A small proportion of primary cells was directly stained against desmin following the protocol for immunohistochemistry and fluorescence activated cell sorted (FACSCalibur, BD) to determine the proportion of muscle progenitors.

Trans-well invasion assays were carried out as follows: invasion chambers (8 μm pore size, HTS FluoroBlok Insert, BD Biosciences) were pre-coated overnight with 10 μg/ml rat tail collagen I (Millipore). Lower storage compartments were filled with either control medium alone (DMEM and 10% FBS), or with Slit1 (400 ng/ml), Slit2 (400 ng/ml), Slit1 and Slit2 (400 ng/ml each), or Sema3a (500 ng/ml). Invasion chambers were inserted into the lower storage slot and 2.5×10^3^ myoblasts were seeded in the upper compartment in control medium and incubated for 24 h. Afterwards, membranes were cut out and mounted with DAPI on glass slides. For data analysis, the ratio of invaded to non-invaded cells was calculated for four independent experiments.

Time-lapse 3D chemotaxis assays were performed in a linear gradient chemotaxis μ-slide chamber (Ibidi). Primary cells at a concentration of 2000 cells/μl were mixed together with collagen I and 10 mM NaOH in order to obtain a final suspension of 1000 cells/μl in a 1 mg/ml collagen I gel matrix. The cell–collagen suspension was injected directly into the chemotaxis channel and allowed to polymerize for 3 h. Subsequently, reservoirs were filled with either control medium (DMEM and 10% FBS) or Slit1 (400 ng/ml), Slit2 (400 ng/ml), Slit1 and Slit2 (400 ng/ml each), or Sema3a (500 ng/ml) in control medium. Cell migration was imaged every 15 min for 24 h at 37°C and 5% CO_2_ using a Zeiss Axiovert microscope. Cells were tracked manually with the MTrackJ plugin for ImageJ (Meijering et al., 2012), evaluated using the chemotaxis and migration tool (Ibidi), and shown as center of mass of the total cell population. Cells which did not migrate more than 25 µm were determined as non-migrating.

### Statistics

For all experiments and time points, a minimum of three mutants and littermate controls were evaluated. Cell culture experiments were repeated at least three times in triplicates. A *P*-value of 0.05 or lower was considered significant. For statistical analysis, either a Mann–Whitney-*U* or a two-tailed *t*-test was performed.
